# Melflufen: A Peptide–Drug Conjugate for the Treatment of Multiple Myeloma

**DOI:** 10.3390/jcm9103120

**Published:** 2020-09-27

**Authors:** María-Victoria Mateos, Joan Bladé, Sara Bringhen, Enrique M Ocio, Yvonne Efebera, Luděk Pour, Francesca Gay, Pieter Sonneveld, Joachim Gullbo, Paul G. Richardson

**Affiliations:** 1 Hospital Clínico Universitario de Salamanca/IBSAL/CIC, 37007 Salamanca, Spain; mvmateos@usal.es; 2 Hematology Department, Institut d’Investigacions Biomèdiques August Pi I Sunyer, Hospital Clinic, 08036 Barcelona, Spain; jblade@clinic.cat; 3 Myeloma Unit, Division of Hematology, University of Torino, Azienda Ospedaliero-Universitaria Città della Salute e della Scienza di Torino, 10126 Torino, Italy; sarabringhen@yahoo.com (S.B.); fgay.cittadellasalute@gmail.com (F.G.); 4 University Hospital Marqués de Valdecilla (IDIVAL), University of Cantabria, 39008 Santander, Spain; ocioem@unican.es; 5 Division of Hematology, Department of Internal Medicine, The Ohio State University Comprehensive Cancer Center, Columbus, OH 43210, USA; yvonne.efebera@osumc.edu; 6 Department of Internal Medicine, Hematology and Oncology, University Hospital Brno, 62500 Brno, Czech Republic; Pour.Ludek@fnbrno.cz; 7 Erasmus MC Cancer Institute, 3075 EA Rotterdam, The Netherlands; p.sonneveld@erasmusmc.nl; 8 Department of Medical Sciences, Division of Clinical Pharmacology, Uppsala University, 751 85 Uppsala, Sweden; Joachim.Gullbo@medsci.uu.se; 9 Jerome Lipper Multiple Myeloma Center, Dana-Farber Cancer Institute, Harvard Medical School, Boston, MA 02215, USA

**Keywords:** melflufen, melphalan flufenamide, peptide–drug conjugate, multiple myeloma, new drugs, drug combinations

## Abstract

Despite the availability of new therapies that have led to improved outcomes for patients with multiple myeloma, most patients will eventually relapse. With triplet and even quadruplet combination therapies becoming standard in the first and second line, many patients will have few treatment options after second-line treatment. Melflufen (melphalan flufenamide) is a first-in-class peptide–drug conjugate (PDC) that targets aminopeptidases and rapidly releases alkylating agents into tumor cells. Once inside the tumor cells, melflufen is hydrolyzed by peptidases to release alkylator molecules, which become entrapped. Melflufen showed anti-myeloma activity in myeloma cells that were resistant to bortezomib and the alkylator melphalan. In early phase studies (O-12-M1 and HORIZON [OP-106]), melflufen plus dexamethasone has demonstrated encouraging clinical activity and a manageable safety profile in heavily pretreated patients with relapsed/refractory multiple myeloma, including those with triple-class refractory disease and extramedullary disease. The Phase III OCEAN study (OP-104) is further evaluating melflufen plus dexamethasone in patients with relapsed/refractory multiple myeloma. The safety profile of melflufen is characterized primarily by clinically manageable hematologic adverse events. Melflufen, with its novel mechanism of action, has the potential to provide clinically meaningful benefits to patients with relapsed/refractory multiple myeloma, including those with high unmet needs.

## 1. Introduction

Despite improved outcomes in patients with multiple myeloma following the advent of proteasome inhibitors, immunomodulatory agents (IMiDs), and anti-CD38 monoclonal antibodies, the majority of patients with multiple myeloma will eventually relapse [1,2]. For younger, fit patients with multiple myeloma, the current frontline therapy includes a proteasome inhibitor plus dexamethasone in triplet combination, often with an IMiD such as thalidomide or lenalidomide, followed by autologous stem cell transplant and lenalidomide maintenance therapy [3,4,5]. Quadruplet regimens consisting of anti-CD38 monoclonal antibodies in combination with an IMiD, a proteasome inhibitor, and a steroid in frontline therapy are also used in some patients [6,7,8]. For patients with newly diagnosed multiple myeloma who are not eligible for stem cell transplant, several multi-agent regimens are recommended. Most of these regimens are based on bortezomib plus dexamethasone or lenalidomide plus dexamethasone as a backbone [9,10,11,12,13]. Several new combinations that have been approved in first-line therapy for patients who are not eligible to receive a stem cell transplant include triplet combination regimens with daratumumab plus lenalidomide and dexamethasone as well as quadruplet regimens with daratumumab plus bortezomib in combination with melphalan and prednisone or thalidomide and dexamethasone [8,14].

Treatment choice following relapse is largely dependent on prior received therapy and prior response to therapy, with class switching often prioritized [15]. Because multiple myeloma is a heterogeneous disease [16] and sequential therapeutic intervention is required to maintain disease control, additional mutations develop throughout the course of the disease, many of which drive resistance to therapy [17,18]. Furthermore, patients are receiving several drug classes during upfront therapy and the use of newer drugs has moved to earlier lines of therapy, resulting in many patients being faced with disease that is refractory to multiple drug classes and multiple drugs within each class after second-line therapy [2,3,15]. Importantly, treatment duration and time to disease progression get progressively shorter with subsequent lines of therapy, while the frequency of toxicities and comorbidities become higher [19].

Thus, new therapies with novel mechanisms of action that are also tolerable are needed for third-line treatment and beyond, particularly for patients with relapsed and/or refractory multiple myeloma who have disease that is refractory to standard-of-care agents including IMiDs, proteasome inhibitors, and anti-CD38 antibodies. Several new agents with novel mechanisms of action are currently under investigation.

The US Food and Drug Administration granted accelerated approval to selinexor in July 2019, a selective inhibitor of exportin 1, in combination with dexamethasone for the treatment of adult patients with relapsed/refractory multiple myeloma who have received ≥4 prior lines of therapy and whose disease is penta-refractory (i.e., ≥2 proteasome inhibitors, ≥2 IMiDs, and ≥1 anti-CD38 monoclonal antibody) [20]. Approval was based on the results from the STORM study, a Phase II study of selinexor plus dexamethasone in heavily pretreated patients with triple-class refractory multiple myeloma (refractory to an IMiD, a proteasome inhibitor, and an anti-CD38 monoclonal antibody) [21].

Venetoclax, a Bcl-2 inhibitor, has shown promising efficacy and an acceptable safety profile when given in combination with bortezomib and dexamethasone in patients with relapsed/refractory multiple myeloma in a Phase Ib study (*n* = 66) [22]. Additional studies are ongoing [23]. Another mechanism of action being explored is the targeting of B cell maturation antigen (BCMA), which is an antigen with expression that is primarily restricted to late stages of B cell differentiation (e.g., late memory B cells and plasma cells) and that is also expressed at high levels in malignant multiple myeloma cells [24]. Immunotherapies targeting BCMAs include anti-BCMA antibody–drug conjugates, bispecific antibodies, bispecific T cell engagers (BiTEs), and chimeric antigen receptor (CAR) T cells [24,25,26]. In the Phase II DREAMM-2 study, belantamab mafodotin, an anti-BCMA antibody–drug conjugate, has demonstrated single-agent activity and a manageable safety profile in patients with relapsed/refractory multiple myeloma who had received ≥3 lines of therapy and were refractory to an IMiD, a proteasome inhibitor, and an anti-CD38 antibody (*n* = 196) [26]. The overall response rate (ORR) was 33% across two different doses of belantamab mafodotin (2.5 and 3.4 mg/kg) and the median progression-free survival (PFS) was 3 months with a 2.5 kg/mg dose and 5 months with a 3.4 kg/mg dose with a short duration of follow-up (median: 6.3 and 6.9 months, respectively). Hematologic events (thrombocytopenia, 27%; anemia, 23%) and keratopathy (24%) were the most commonly reported Grade 3/4 adverse events (AEs) [26]. Several types of CAR T cells have been evaluated in a total of >300 patients with relapsed/refractory multiple myeloma in early phase studies [25]. CAR T cells have shown promising clinical activity with high response rates (ranging from 20–100%; most were ≥80%) in these Phase I studies [25]. For example, in a Phase I study, idecabtagene vicleucel (bb2121) showed an 85% ORR among 33 evaluable patients with a median duration of response of 10.9 months and a median PFS of 11.8 months. Hematologic events were the most common Grade 3/4 AEs (neutropenia, 85%; leukopenia, 58%; anemia, 45%; thrombocytopenia, 45%) [27].

Melphalan flufenamide (melflufen) is a first-in-class peptide–drug conjugate (PDC) that targets aminopeptidases and rapidly releases alkylating agents into tumor cells [28,29,30,31,32]. Because of its distinct mechanism of action, melflufen is well suited for use in third-line therapy both in patients who have not received prior alkylator therapy, such as older patients and those who are not eligible for transplant, as well as in patients who have prior exposure to melphalan [29]. This review describes the historical development of melflufen, its mechanism of action, and the ongoing clinical development program for melflufen in multiple myeloma.

## 2. Preclinical Development

### 2.1. Mechanism of Action

Melflufen is a PDC that is being investigated in multiple myeloma and other hematologic malignancies, immunoglobulin light chain (AL) amyloidosis, and solid tumors [29,33,34].

**Figure 1:**
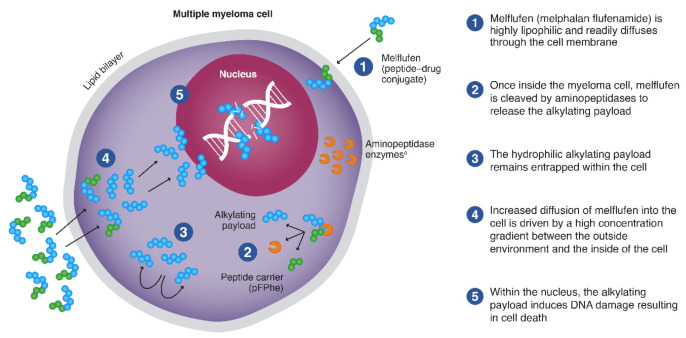
Mechanism of action of melflufen. Melflufen readily diffuses through the cell membrane into the myeloma cell, where it is cleaved by aminopeptidases to release the alkylating payload that are entrapped. ^a^ Aminopeptidases are proteolytic enzymes expressed in cancer cells, including multiple myeloma cells. pFPhe, p-L fluoro-phenylalanine ethyl ester. From Oriol A, et al. *Expert Opin Invest Drugs*. 2020 Sep 14 [online ahead of print] doi: 10.1080/13543784.2020.1808884. Copyright © 2020 Taylor & Francis. Reprinted with permission from Taylor & Francis Ltd.

Melflufen is highly lipophilic, which promotes its rapid uptake by cells [29,30,31,35] (Figure 1). Once within the cell, melflufen releases its hydrophilic alkylator payloads via the hydrolytic activity of intracellular peptidases (e.g., aminopeptidases) [30]. Aminopeptidases are Zn^2+^-dependent metalloproteinases that remove amino acids at the *N*-terminal position from oligopeptides and have been associated with multiple tumorigenic processes such as proliferation, apoptosis, differentiation, angiogenesis, and motility [36,37].

The dependence of melflufen on aminopeptidases was initially demonstrated by the reduced cytotoxic activity of melflufen—but not the alkylator melphalan—when cells were pretreated with bestatin, an antibiotic that is a potent aminopeptidase inhibitor [31]. In addition, structure analogs designed to resist peptide hydrolysis (*N*-methyl derivative and derivative with d-amino acid) were shown to be almost 100-fold less potent than melflufen [31]. Subsequent in vitro studies demonstrated that hydrolytic cleavage of melflufen by aminopeptidases releases alkylator payloads, including melphalan [30]. In vitro, the activity of melflufen is multi-pronged, including induction of DNA damage, induction of apoptosis, inhibition of VEGF-dependent cell migration, and inhibition of tumor-associated angiogenesis, which have been further reviewed elsewhere [29]. Downregulation of aminopeptidases resulted in reduced melflufen-mediated cytotoxic activity and apoptotic signaling in cultured cells [30].

### 2.2. Preclinical Anti-Tumor Activity

The anti-tumor activity of melflufen in multiple primary cultures of human cancer and leukemia cells, as well as established cell lines, was first reported in 2003 [31,33]. Compared with the known alkylator melphalan, melflufen had a higher cytotoxic activity in this broad range of malignant human cells, with a mean IC_50_ value that was 35-fold lower with melflufen than melphalan [29] (Table 1). In cells from hematological malignancies, the higher potency of melflufen compared with melphalan was even more pronounced (mean IC_50_ values were ≈50-fold lower with melflufen) [28,29,38,39]. Primary cultures of patient-derived acute myeloid leukemia cells were 7-fold more sensitive than normal peripheral blood mononuclear cells, indicating a 7-fold in vitro therapeutic index [39].

Melflufen also has a higher lipophilicity than melphalan, and peak intracellular concentration of melphalan can be achieved much faster with melflufen than melphalan [30,33,40]. More recently, the anti-tumor activity of melflufen has been reported in ovarian cancer, breast cancer, osteosarcoma, acute myeloid leukemia, neuroblastoma, and multiple myeloma cell lines [28,39,41,42,43,44]. In addition, melflufen demonstrated anti-tumor activity in a multiple myeloma xenograft model [28] and a genetically engineered myeloma model in transgenic immunocompetent Vk*MYK mice, postulated to be predictive of clinical activity [45].

Melflufen is rapidly taken up into cells, with the maximum concentration of intracellular melphalan reached within 15 min and full therapeutic activity obtained after 30 min of exposure in vitro, which is faster than that of melphalan [30,31]. In multiple myeloma cells, a high concentration of intracellular melphalan can be reached with a lower dose of drug (5 μM melflufen vs. 100 μM melphalan) [28]. In patient-derived myeloma tumor samples, melflufen demonstrated ≈50-fold higher cytotoxicity than melphalan, and a 50-fold higher melphalan exposure than direct administration of melphalan [42]. Melflufen has demonstrated anti-tumor activity against multiple myeloma cells that show resistance to melphalan, bortezomib, and dexamethasone. This activity likely stems from the multiple downstream effects elicited by melflufen, including induction of apoptosis and triggering rapid, robust, and irreversible DNA damage [28,32]. Unlike with melphalan, the DNA damage induced by melflufen is not dependent on activation of p53 [28], which likely contributes to the activity of melflufen in melphalan-resistant cells. In patients with multiple myeloma who have the 17p13 [del(17p)] adverse risk genotype, mutations/deletions in *TP53* are found in approximately one-third of newly diagnosed patients and at least 50% of those with relapsed and refractory disease. Mutations in *TP53* confer a poor prognosis and are associated with resistance to therapy [46].

Melflufen has also demonstrated synergistic activity when combined with standard-of-care agents in myeloma such as dexamethasone in dexamethasone-sensitive multiple myeloma cell lines [28] and bortezomib and lenalidomide in cell lines that were resistant to standard-of-care drugs [28,42]. Melflufen has anti-angiogenic activity and inhibits cell migration in multiple myeloma cells [28]. The potent anti-angiogenic effect of melflufen has been demonstrated in multiple in vitro and in vivo models [47]. There is some evidence that melflufen can also overcome the cytoprotective effects of the bone marrow microenvironment [28]. These lines of evidence show that (1) high intracellular concentrations of alkylator can be achieved with melflufen; (2) melflufen is highly cytotoxic and more potent than melphalan; (3) melflufen has broad anti-tumor activity in multiple myeloma with no apparent cross-resistance to other drugs; and (4) melflufen shows synergistic activity with standard- of-care agents, and together support the potential for melflufen as a myeloma therapy.

## 3. Pharmacokinetics

The pharmacokinetics of melflufen were first evaluated in an open-label, multicenter, dose-finding Phase I/II study in patients with solid tumors [48]. A total of 29 patients were evaluated for pharmacokinetics. Most patients received 50 mg melflufen, but doses ranged from 25 to 130 mg. Melflufen and melphalan (resulting from aminopeptidase cleavage of melflufen) concentrations were assessed before the start of infusion and then at several intervals up to 360 min after the start of the infusion, including at the end of infusion, which occurred at 30 min. Among patients who received 50 mg melflufen, the peak plasma concentration (C_max_) of melflufen was generally observed right before the end of infusion, whereas the C_max_ of melphalan was observed 5 to 15 min after the end of infusion (Table 2). The release of melphalan following infusion with melflufen was rapid, as suggested by the fact that the C_max_ and area under the curve over the time of infusion (AUC_0–0.5_) were higher for melphalan than melflufen. The elimination and clearance of melphalan was not affected by body weight or sex.

BRIDGE (OP-107) is a Phase II study evaluating the pharmacokinetics of melphalan during treatment with melflufen and dexamethasone in patients with relapsed/refractory multiple myeloma and moderate to severely impaired renal function (NCT03639610). Given that renal impairment is a common complication in patients with multiple myeloma, occurring in ≈50% of patients with multiple myeloma [49], the results from BRIDGE will be of relevance for patients with multiple myeloma and renal insufficiency. Because melflufen is rapidly and completely metabolized by aminopeptidases and melphalan is primarily eliminated from the plasma by spontaneous hydrolysis [31,50]—a process independent of renal function and hepatic metabolism—the hypothesis is that renal impairment will have no effect on melflufen pharmacokinetics and only a minor effect on melphalan pharmacokinetics.

## 4. Clinical Development

### 4.1. Early Development in Multiple Myeloma

Melflufen was first evaluated clinically in multiple myeloma in O-12-M1 (NCT01897714), a Phase I/II, multicenter, dose-escalation and dose-expansion study of melflufen with or without dexamethasone in patients with relapsed/refractory multiple myeloma who had received ≥2 prior lines of therapy, including lenalidomide and bortezomib, and were refractory to the last line of therapy [51]. A total of 75 heavily pretreated patients were enrolled in the study. In the Phase I dose-finding portion of the study, melflufen was administered intravenously over 30 min on Day 1 of each 21-day cycle. A total of 4 four dose levels of melflufen (15, 25, 40, and 55 mg) were assessed. At the 55 mg dose, 4 of 6 patients experienced Grade 4 dose-limiting hematologic toxicities. Therefore, the recommended dose for expansion was 40 mg melflufen. Among 58 patients treated at the recommended dose in the Phase II portion of the study, 13 received single-agent melflufen and 45 received melflufen in combination with 40 mg dexamethasone weekly. Of the 45 patients who received the combination therapy, 28 initiated treatment with 21-day cycles, but the Data Safety Monitoring Committee recommended increasing the cycle length to 28 days to prolong the hematologic recovery time between cycles. An additional 17 patients started treatment with a 28-day cycles. Among the 45 patients who received the combination therapy, the median number of prior therapies was 4 (range, 2–14) and 67% of patients were refractory to a proteasome inhibitor and an IMiD. The ORR (≥partial response (PR)) was 31%, with 5 patients achieving a very good PR (VGPR) and 9 patients achieving a PR (Table 3) with a median duration of response of 8.4 months [51]. The ORR was 41% among patients who received ≥2 doses of study treatment and had a post-baseline response assessment (*n* = 34). Responses (≥minimal response) were also observed in 4 of 9 patients with melphalan-refractory disease. At a median follow-up of 27.9 months, the median PFS was 5.7 months and the median overall survival (OS) was 20.7 months. In a subsequent analysis, with a median follow-up of 46 months, the median OS was also 20.7 months [52].

Melflufen plus dexamethasone was generally manageable in this heavily pretreated patient population [51]. All patients experienced ≥1 AE, most commonly hematologic AEs including thrombocytopenia (73%), neutropenia (69%), and anemia (64%). The most common non-hematologic AEs included pyrexia (40%), asthenia (31%), fatigue (29%), nausea (27%), and diarrhea (24%). Melflufen-related Grade ≥3 AEs occurred in 82% of patients, most commonly reversible thrombocytopenia and neutropenia. The incidence of Grade 4 thrombocytopenia was reduced from 32% to 0%, and the median duration of study treatment increased (from 105 to 182 days) after the study cycle was lengthened from 21 to 28 days. The most common Grade 3/4 non-hematologic AEs were asthenia, pneumonia, and hyperglycemia, and C-reactive protein increase (7% each). Serious AEs (SAEs) occurred in 38% of patients and were considered by the investigator to be related to melflufen in 27% of patients. The most common SAE was pneumonia. Overall, the safety profile of melflufen plus dexamethasone is generally comparable to that of other doublet combinations in patients with heavily pretreated relapsed/refractory multiple myeloma [53,54], with hematologic AEs being the most frequently reported AEs. In addition, gastrointestinal AEs (e.g., nausea, vomiting, diarrhea) and pyrexia are among the most frequently observed non-hematologic AEs in these patients. Pneumonia, which was also reported with melflufen plus dexamethasone (16% overall; 7% Grade 3/4), has also been frequently reported with other regimens, including pomalidomide plus dexamethasone (16%; Grade 3/4, 11%) and bortezomib plus dexamethasone (13%; Grade 3/4, 11%). Peripheral neuropathy, an AE frequently observed with bortezomib plus dexamethasone (67%; Grade 3/4, 15%), was not commonly observed with melflufen [53,54]. Among patients who received melflufen plus dexamethasone (*n* = 45), 10 received at least 8 cycles of therapy and 35 discontinued treatment before 8 cycles of therapy, most commonly due to AEs (*n* = 18) and disease progression (*n* = 13). Of the 18 patients who discontinued the combination therapy due to AEs, 16 (89%) had received the 21-day regimen and 2 (11%) the 28-day regimen [51].

Among the 13 patients who received single-agent melflufen, the median number of prior therapies was 5 (range, 4–8), the ORR was 8% (1 PR), median PFS was 4.4 months, and median OS was 15.5 months. Of note, patients treated with single-agent melflufen appeared to have a more advanced disease than those treated with melflufen plus dexamethasone (e.g., median years since diagnosis: 8 vs. 5; median prior lines of therapy: 5 vs. 4; and prior daratumumab exposure: 46% vs. 13%, respectively). Overall, results from the single-agent group are comparable to those of other studies being conducted at the time in similar patient populations, including studies of pomalidomide plus dexamethasone and daratumumab alone [53,55]. However, due to a better efficacy signal in the combination cohort, the single-agent arm of the study was terminated early. Results from the melflufen plus dexamethasone combination arm of the O-12-M1 study supported the further development of melflufen in combination with dexamethasone, including evaluation in potential triplet combination regimens.

### 4.2. Efficacy and Safety of Melflufen Combination Therapies

HORIZON (OP-106; NCT02963493), a pivotal, single-arm, multicenter Phase II study evaluating the efficacy and safety of melflufen in combination with dexamethasone, demonstrated efficacy and a manageable safety profile for the doublet in patients with heavily pretreated and poor-risk relapsed/refractory multiple myeloma refractory to pomalidomide and/or an anti-CD38 monoclonal antibody in an interim analysis (data cutoff date 1 October 2019) (Table 3 and Table 4) [56]. Of 154 patients who had received study treatment at the time of the data cutoff, all patients had prior exposure to IMiDs and proteasome inhibitors, 79% had prior exposure to anti-CD38 monoclonal antibodies, 71% were triple-class refractory, and 97% were refractory to treatment in the last line. The median treatment duration was 14.3 weeks.

Among 125 patients evaluable for response, the ORR (≥PR) was 29%, with 1 patient achieving a stringent complete response (CR) and 10 patients achieving a VGPR. The median duration of response was 4.4 months. The ORR was 21% among 47 patients with high-risk cytogenetics, 24% among 93 patients with triple-class refractory disease, and 24% among 42 patients with extramedullary disease. The median PFS and OS were 4.2 and 11.6 months for all patients, 4.0 and 11.3 months for patients with triple-class refractory disease, and 3.0 and 8.1 months for patients with extramedullary disease, respectively. Overall, 97% of patients experienced any-grade AEs and 85% of patients experienced Grades 3/4 AEs, most commonly hematologic AEs (thrombocytopenia [69%], neutropenia [66%], and anemia [37%]). The most common (occurring in ≥5% of patients) SAEs and treatment-related SAEs were infections (19% and 5%), febrile neutropenia (5% and 5%), and thrombocytopenia (5% and 5%). A total of 108 patients (70%) had discontinued treatment as of the data cutoff, 73 (47%) due to disease progression and 21 (14%) due to AEs. The rate of treatment discontinuation due to AEs in HORIZON was similar or lower than those reported for other doublet combination therapies, including pomalidomide plus dexamethasone (6%), bortezomib plus dexamethasone (20%), and selinexor plus dexamethasone (33%) [21,53,54]. Overall, 5 deaths were reported in the study, with none deemed to be related to melflufen. In general, the safety profile of melflufen plus dexamethasone in HORIZON was consistent with that reported in O-12-M1.

ANCHOR (OP-104; NCT03481556) is a Phase I/II study evaluating the safety and efficacy of melflufen and dexamethasone in triplet combinations with daratumumab or bortezomib in patients with relapsed/refractory multiple myeloma [57]. Eligible patients had to have received 1–4 prior lines of therapy and be refractory to an IMiD and/or a proteasome inhibitor (only applies to patients in the daratumumab cohort). In an interim analysis of the ANCHOR study (data cutoff date 8 October 2019), the triplet combinations of melflufen, dexamethasone, and daratumumab or bortezomib showed encouraging clinical activity and no new safety signals (Table 3 and Table 4). In the dose-escalation portion of the study, patients received 1 of 2 doses of melflufen (30 or 40 mg) on Day 1 of each 28-day cycle.

In the daratumumab cohort (combined Phase I/II), patients received melflufen plus dexamethasone (40 mg) plus 16 mg/kg daratumumab [57]. Of 33 patients treated up to the data cutoff date, 6 received 30 mg melflufen and 27 received 40 mg melflufen. Most patients (88%) had prior exposure to alkylator therapy and 4 patients (12%) were refractory to alkylator therapy. At a median follow-up of 6.6 months, the median duration of treatment was 6.2 months, and 67% of patients remained on study treatment. No dose-limiting toxicities (DLTs) were reported. The ORR (≥PR) was 76%, with 1 patient achieving a stringent CR and 11 patients achieving a VGPR, and the median PFS was 14.3 months. With 30 and 40 mg melflufen, Grade 3/4 treatment-related AEs, most commonly hematologic AEs, were reported in 83% and 81% of patients, respectively. Overall, SAEs were reported in 36% of patients and 3 patients died due to progressive disease, including 1 patient who had Grade 5 sepsis and pneumonia while in progression.

In the bortezomib cohort (Phase I only), patients received melflufen plus dexamethasone (20–40 mg) plus 1.3 mg/m^2^ bortezomib. Of 6 patients treated up to the data cutoff date, 3 received 30 mg melflufen and 3 received 40 mg melflufen. All patients had received prior proteasome inhibitor therapy and 5 had received prior alkylator therapy. At a median follow-up of 13.4 months, the median duration of treatment was 9.3 months, and 50% of patients remained on the study treatment. No DLTs were reported. The ORR (≥PR) was 67%, with 2 patients achieving VGPR, and the median PFS was not reached. The most common AEs and Grade 3/4 treatment-related AEs were hematologic events that were clinically manageable. In total, SAEs were reported in 5 patients and 2 patients died due to disease progression after discontinuation of study treatment.

## 5. Additional Clinical Development

The preliminary data from the HORIZON study, demonstrating encouraging clinical efficacy and a manageable safety profile for melflufen plus dexamethasone [56], support the further development of this combination. Based on clinical data to date, OCEAN (OP-103; NCT03151811)—a randomized, head-to-head, superiority, open-label, global Phase III study of melflufen plus dexamethasone versus pomalidomide plus dexamethasone in patients with multiple myeloma who have received 2 to 4 prior therapies, including lenalidomide within 18 months and are refractory to last line of therapy—was initiated (Table 4) [58,59].

In addition, the combination of melflufen and dexamethasone is also being evaluated in a Phase I/II study (OP201; NCT04115956) in patients with AL amyloidosis [34], which is a rare neoplastic disease of the plasma cells that results in the accumulation of aggregates of misfolded immunoglobulin free light chains within vital organs, leading to organ damage [60]. Despite treatments commonly used in multiple myeloma also being used in patients with AL amyloidosis, there are currently no approved therapies for this patient population with a high unmet medical need [60,61,62].

## 6. Management of Melflufen

Melflufen (40 mg) is given as a 30-min central intravenous infusion (Day 1 of a 28-day cycle) in combination with 40 mg oral dexamethasone (Days 1, 8, 15, and 22) [51]. Peripheral administration of melflufen will be investigated to enable treatment of patients without central venous access.

Given that neutropenia and thrombocytopenia are the most common toxicities with melflufen, monitoring for these cytopenias and providing appropriate management and supportive care are recommended [63]. During the O-12-M1 study, the cycle length was modified from 21 to 28 days to allow for hematologic recovery [51]. In addition, dose modifications of melflufen due to hematologic toxicities were permitted. If patients did not meet the hematologic criteria for beginning a new cycle (absolute neutrophil count ≥1.0 × 10^9^/L; platelet count ≥50.0 × 10^9^/L) by Day 1 of the next cycle, patients were re-evaluated weekly. If criteria for initiation were met on Day 29 or 36, no dose adjustments were needed; if they were met on Day 43, a one-level dose reduction (from 40 mg to 25 mg) could occur at the investigator’s discretion. If the criteria for initiation were met on Day 50 or 57, a one-level dose level reduction was required. An additional week could be added to the cycle length of subsequent cycles, at the investigator’s discretion, but treatment had to be initiated by Day 42 [51]. Additional management strategies include growth factor support, platelet transfusions, and the use of romiplostim (US only) [63,64].

## 7. Conclusions

Given that current standard-of-care regimens utilize triplet and quadruplet combination regimens in the first and second lines, there will likely be a large unmet need for patients with multiple myeloma that is refractory to multiple agents in the second line and beyond [2,3,15]. Importantly, with more treatments, patients will have progressively shorter duration of response to each subsequent therapy [19]. The novel agent melflufen, a PDC, has the potential to fill an unmet clinical need in the multiple myeloma treatment landscape. Melflufen has a novel mechanism of action and has demonstrated preclinical and clinically meaningful activity in multiple myeloma, which is refractory to prior standard-of-care therapies. In the early Phase O-12-M1 and HORIZON studies, melflufen plus dexamethasone showed efficacy and a manageable safety profile in patients with heavily pretreated relapsed/refractory multiple myeloma, including patients with triple-class refractory multiple myeloma and those with extramedullary disease [51,52,56]. Importantly, 83% and 57% of patients in HORIZON had been exposed to and were refractory to alkylators, respectively [56], which suggests that melflufen is efficacious in patients who are refractory to alkylators, and this is supported by the preclinical data indicating that melflufen has activity in multiple myeloma cells that are resistant to melphalan [28]. This would not be surprising given that melflufen has three factors that distinguish it from melphalan: (1) melflufen is lipophilic and can be rapidly taken up by myeloma cells; (2) melflufen can achieve higher intracellular concentrations of drug more rapidly than melphalan; and (3) melflufen has ≈50-fold higher cytotoxicity than melphalan in patient-derived myeloma tumor samples [28,29,32,42].

To date, the safety profile of melflufen has been consistent across studies, and no new safety concerns have been identified when melflufen is administered in doublet and triplet combinations [51,56,57]. In general, hematologic events that are manageable have been identified as the most common toxicity, which is not surprising because hematologic toxicities are commonly reported with other investigational agents in heavily pretreated patients with relapsed/refractory multiple myeloma [21,65,66].

Taken together, these data suggest that melflufen plus dexamethasone, as a doublet and in combination with other drugs (daratumumab, bortezomib), has the potential to be beneficial for a broad range of patients with relapsed/refractory multiple myeloma in third- or even second-line therapy. Additionally, melflufen could also have a potential role as a conditioning regimen in patients with relapsed/refractory multiple myeloma who are eligible for stem cell transplant [67].

## Figures and Tables

**Table 1:** Comparison between melflufen and the known alkylator melphalan [30,33,40].

Characteristic	Melflufen	Melphalan
Chemical structure	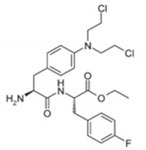	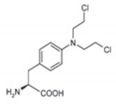
Peak intracellular melphalan concentration *	Relative concentration: ≈80 (achieved after 15 min exposure)	Relative concentration: ≈4 (achieved after 60 min exposure)
Mean IC_50_, μM ^†^	0.05	1.5
Lipophilicity (LogP) ^‡^	4.0	−0.97

* Evaluated in U1810 cells after up to 120 min of exposure with 10 μM melflufen and 10 μM melphalan. ^†^ Mean IC_50_ value was obtained from 15 primary cultures from patients with hematologic malignancies. ^‡^ The partition coefficient, LogP, is a measure of the propensity for a compound to dissolve in lipids and water. A positive value indicates a higher concentration in the lipid phase, whereas a negative value indicates a higher concentration in water.

**Table 2:** Pharmacokinetics of melflufen and melphalan following administration with 50 mg melflufen [48].

Parameter, Median (Range) *	Melflufen	Melphalan
C_max_, ng/mL	176 (43–1306)	513 (320–1455)
t_max_, h	0.42 (0.32–0.58)	0.58 (0.42–1.48)
AUC_0–0.5_, ng/mL*h	40.3 (11.8–162)	92.2 (41.1–181)
AUC_0–∞_, ng/mL*h	-	895 (511–1503)
t_1/2_, h	-	1.09 (0.83–1.83)
Clearance, L/h	-	32.0 (19.0–56.0)

* Evaluated in patients with solid tumors, including ovarian cancer, non-small cell lung cancer, gastrointestinal cancer, and breast cancer who received 50 mg melflufen as a 30 min infusion. Samples were collected at baseline (prior to the start of infusion) and at 20, 25, 30, 35, 45, 60, 90, 120, 240, and 360 min after the start of infusion. AUC, area under curve; C_max,_ maximum concentration; t_1/2_, time for C_max_ to halve; t_max_, time to reach C_max_.

**Table 3:** Efficacy of melflufen combination therapies in patients with multi-refractory multiple myeloma [51,52,56,57].

Study	Treatment	Patients, *n*	Prior Therapies, Median (Range)	Median Follow-Up, mo	ORR, %	Median PFS (95% CI), mo	Safety Profile
O-12-M1 (Phase I/II)	Melflufen + dexamethasone; median duration:4.1 mo	45 (Phase II; evaluable for response)	4 (2–14)	27.9	31	5.7 (3.7–9.2)	Any-grade AEs: 100% Melflufen-related Grade ≥3 AEs: 82% Study cycle length increase allowed for full hematologic recovery and reduced the frequency of Grade 4 thrombocytopenia SAEs: 38% Treatment-related SAEs: 27% Most common nonhematologic AEs (≥20%): pyrexia, asthenia, fatigue, nausea, and diarrhea Most common treatment-related SAE (≥5%): pneumonia (9%)
HORIZON * (Phase II)	Melflufen + dexamethasone; median duration: 14.3 wk	154 (125 evaluable for response)	5 (2–12)	Not reported	29	4.2 (3.7–4.9)	Any-grade AEs: 97% Grades 3/4 AEs: 85% Treatment-related SAEs (≥5%): infections and infestations (5%), febrile neutropenia (5%)
ANCHOR ^†^ (Phase I/II)	Melflufen (30 or 40 mg) + dexamethasone + daratumumab; median duration: 6.2 mo	33 (30 mg, *n* = 6; 40 mg, *n* = 27)	30 mg: 2.5 (1–3) 40 mg: 2.0 (1–4)	6.6	76	14.3 (9.7-NR)	No DLTs Treatment-related Grade 3/4 AEs: 82% (83% and 81%) SAEs: 36% Treatment-related SAEs: 18% Treatment-related SAEs (≥5%): febrile neutropenia (6%)
	Melflufen (30 or 40 mg) + dexamethasone + bortezomib; median duration: 9.3 mo	6 (30 mg or 40 mg, *n* = 3 each)	2.5 (2–4)	13.4	67	NR	No DLTs SAEs: 83% Treatment-related SAEs: 17% Treatment-related SAEs: pneumonia and neutropenia (*n* = 1)

* Patients received 40 mg melflufen (Day 1) plus 40 mg dexamethasone (20 mg if aged ≥75 years) weekly (Days 1, 8, 15, and 22) of each 28-day cycle. ^†^ In the daratumumab arm, patients received melflufen (Day 1) plus 40 mg dexamethasone weekly (Days 1, 8, 15, and 22 of each cycle and an additional dose on Day 2 of Cycle 1), plus 16 mg/kg daratumumab (Days 2, 8, 15, and 22 in Cycle 1; Days 1, 8, 15, and 22 in Cycle 2; Days 1 and 15 in Cycles 3–6; and Day 1 in Cycle 7 and beyond). In the bortezomib arm, patients received melflufen (Day 1) plus dexamethasone (20 mg on Days 1, 4, 8, and 11; and 40 mg on Days 15 and 22), plus 1.3 mg/m^2^ bortezomib (Days 1, 4, 8, and 11). AE, adverse event; DLT, dose-limiting toxicity; NR, not reached; ORR, overall response rate (≥partial response); PFS, progression-free survival; SAE, serious AE.

**Table 4:** Ongoing studies of melflufen in multiple myeloma.

Study (NCT ID)	Treatment Arm	Patients	Study Status [59]
HORIZON Phase II (NCT02963493)	Melflufen + dexamethasone	Received ≥2 lines of prior therapy; refractory to pomalidomide and/or anti-CD38 monoclonal antibody	Ongoing (fully enrolled) Enrollment: 157 Primary completion date: June 2020
ANCHOR Phase I/II (NCT03481556)	Melflufen + dexamethasone + daratumumab or bortezomib	Relapsed/refractory; received 1–4 prior lines of therapy	Recruiting Estimated enrollment: 80 Estimated primary completion date: December 2020
OCEAN Phase III (NCT03151811)	Melflufen + dexamethasone vs. pomalidomide + dexamethasone	Relapsed/refractory; received 2–4 prior lines of therapy; refractory to lenalidomide in the last line of therapy	Recruiting Estimated enrollment: 450 Estimated primary completion date: March 2021
